# Development and validation of main spectral profile for rapid identification of *Yersinia ruckeri* isolated from Atlantic salmon using matrix-assisted laser desorption/ionization time-of-flight mass spectrometry

**DOI:** 10.3389/fvets.2022.1031373

**Published:** 2022-10-20

**Authors:** Rasaq A. Ojasanya, Ian A. Gardner, David Groman, Sonja Saksida, Matthew E. Saab, Krishna K. Thakur

**Affiliations:** ^1^Department of Health Management, Atlantic Veterinary College, University of Prince Edward Island, Charlottetown, PE, Canada; ^2^Aquatic Diagnostic Services, Atlantic Veterinary College, University of Prince Edward Island, Charlottetown, PE, Canada

**Keywords:** MALDI-TOF MS, main spectral profile, *Yersinia ruckeri*, aquaculture, validation

## Abstract

Matrix-assisted laser desorption/ionization time-of-flight mass spectrometry (MALDI-TOF MS) allows rapid and reliable identification of microorganisms. The accuracy of bacterial identification using MALDI-TOF MS depends on main spectral profiles (MSPs) provided in a quality-assured commercial reference library, which requires ongoing improvement. This study aimed to develop and validate an in-house MALDI-TOF MS MSP to rapidly identify *Yersinia ruckeri* isolated from Atlantic salmon (*Salmo salar*). The novel MSP was prepared using an isolate of *Y. ruckeri* recovered from Atlantic salmon and confirmed by 16S rRNA gene sequencing. Subsequently, a validation set which comprises 29 isolates of *Y. ruckeri* were examined from three fishes: Atlantic salmon (*Salmo salar*) (*n* = 26), American eel (*Anguilla rostrata*) (*n* = 1), and Atlantic cod (*Gadus morhua*) (*n* = 2). These isolates were randomly selected from the Atlantic Veterinary College, Aquatic Diagnostic Services Bacteriology Laboratory's culture collection to validate the novel MSP. Analytical sensitivity of MALDI-TOF MS using the novel MSP to identify the validation set was 86.2%. Repeatability was assessed by acquiring spectra from 30 different spots of a randomly-selected isolate of *Y. ruckeri*, and analyzed spectra from each spot were compared against the novel MSP. The coefficient of variation was 3.3%. The novel MSP clustered with Bruker MSPs (*n* = 3) of *Y. ruckeri* in the reference library and did not falsely identify any closely related bacteria to *Y. ruckeri*. This study reports the development of a novel MSP of high analytical sensitivity and specificity for rapid identification of *Y. ruckeri* using MALDI-TOF MS.

## Introduction

*Yersinia ruckeri*, the causative agent of enteric redmouth disease (ERM), is a bacterial pathogen that affects all life stages of salmonids as well as other fin-fish species, although the clinical disease is more acute in fry and fingerlings than older life stages (1, 2). Clinically, ERM is characterized by hemorrhagic septicemia, which may ultimately lead to mortality, and cumulative mortality can increase from 25 to 75% in the absence of therapeutic interventions (3, 4). *Y. ruckeri* was first isolated in rainbow trout (*Oncorhynchus mykiss*) but has since been detected in other salmonids such as Atlantic salmon (*Salmo salar*), lake trout (*Salvelinus namaycush*), brown trout (*Salmo trutta*), brook trout (*Salvelinus fontinalis*), Coho salmon (*Oncorhynchus kisutch*), cutthroat trout (*Oncorhynchus clarkii*), sockeye (*Oncorhynchus nerka*), Chinook (*Oncorhynchus tshawytscha*), and other species of fish like common carp (*Cyprinus carpio*), catfish (*Silurus glanis*), goldfish (*Carassius auratus*), European eel (*Anguilla anguilla*), coalfish (*Pollachius virens*), and perch (*Perca fluviatilis*) (5, 6). Over the past two decades across Atlantic Canada, *Y. ruckeri* has been one of the most commonly-detected bacterial pathogens from salmonid samples submitted to the Atlantic Veterinary College, Aquatic Diagnostic Services Bacteriology Laboratory (7).

Early detection of *Y. ruckeri* can hasten intervention and circumvent mortality events during ERM outbreaks in farmed salmonids (8, 9). The traditional method of identifying this pathogen in the laboratory is by culture and isolation using general-purpose media, usually followed by a series of biochemical tests to determine the genus and species of the bacterium (10). The time for identification of this bacterium in the laboratory can be 4 to 7 days if traditional methods are used (11). Biochemical tests are sometimes prone to species-level misidentification because *Y. ruckeri* is phylogenetically and biochemically similar to other species of the genus *Yersinia*. In addition, *Y. ruckeri* can also be misidentified or other members of the order Enterobacterales, such as *Hafnia alvei*, which is a common misidentification whenever *Y. ruckeri* is analyzed using the Analytical Profile Index^®^ biochemical testing system (12, 13). Other *Yersinia* species that have been detected in Atlantic salmon and trout include *Y. enterocolitica, Y*. *intermedia, Y. frederiksenii*, while *Y. kristensenii, Y. bercovieri, Y. mollaretii, Y. rohdei*, and *Y. aldovae* have not been reported in salmonids (14–16). Molecular techniques such as polymerase chain reaction (PCR) and sequencing of genes such as 16S ribosomal RNA (rRNA) provide a higher specificity to differentiate *Yersinia* to species-level. However, these can be expensive or resource-consuming processes (and therefore not routinely available in most clinical laboratories). Molecular techniques can take an additional 5–24 h before species-level identification results are available (17, 18).

Many clinical laboratories increasingly utilize matrix-assisted laser desorption/ionization time-of-flight mass spectrometry (MALDI-TOF MS) as a less expensive and faster analytical technique. MALDI-TOF MS has a high level of accuracy for species-level identification of bacteria that affect human and animal health (19–21). MALDI-TOF MS has replaced or supplemented phenotypic and molecular methods of identifying bacteria in modern-day clinical microbiology (22–24). Identification of bacteria to the species-level using MALDI-TOF MS is achieved by matching the spectral profile of bacterial peptides against existing main spectral profile (MSP) in the reference library (widely used in veterinary laboratories is the commercial MALDI Biotyper^®^ reference library manufactured by Bruker), which can be performed in < 1 h (25, 26). The reliability of the MALDI-TOF MS to correctly identify bacteria largely depends on the presence and quality of MSPs in the available reference library (27). Notwithstanding, bacterial identification sometimes fails even with sufficient reference spectra due to the poor quality of the spectral composition (28). Several research investigations have further augmented or supplemented the commercial reference library with custom MSPs of clinically significant bacteria to extend the existing library and increase MALDI-TOF MS's performance in bacterial identification (29, 30). However, the Bruker reference library was developed using bacterial species isolated from humans (31), so there could be difficulty in applying this reference library to aquatic pathogens (32). Variability in bacterial identification using MALDI-TOF MS is linked to several other factors: sample preparation, quality or type of matrix used, acid concentration in the matrix, presence of surfactant in matrix, the concentration of bacterial cells, and instrument conditions (33). This study aimed to develop and validate an in-house main spectral profile for rapidly identifying *Y. ruckeri* isolated from Atlantic salmon using MALDI-TOF MS.

## Materials and methods

### Bacterial isolates used for the study

Isolates of *Y. ruckeri* from Atlantic salmon (*Salmo salar*) (*n* = 27), Atlantic cod (*Gadus morhua*) (*n* = 2), and American eel (*Anguilla rostrata*) (*n* = 1) were randomly selected from the culture collection of the Atlantic Veterinary College, Aquatic Diagnostic Services Bacteriology Laboratory (AVC ADSBL) at the University of Prince Edward Island. These isolates were primarily obtained during bacteriological analyses of swabs, culture plates, tissues, and fluids samples submitted to AVC ADSBL by aquatic facilities across Atlantic Canada and Ontario. *Y. ruckeri* isolates were identified by Gram stain morphology (gram-negative bacillus), oxidase activity (negative), growth at 35°C (positive), hydrogen sulfide production (negative), and motility (most strains were positive at 22°C). Suspect isolates were confirmed using the Analytical Profile Index^®^ 20E (bioMérieux Inc., NC, USA) and agglutination techniques (34). The electronic laboratory information management system at AVC ADSBL did not document the life-stage data such as fry, parr, smolt grow-out, and brood-stock of submitted samples from which these isolates were recovered.

#### Bacterial culture and sub-culturing of lyophilized isolates

Lyophilized isolates of *Y. ruckeri* were reconstituted using Tryptic Soy Broth (Becton Dickinson and Company, MD, USA) and aseptically plated onto Columbia agar (Oxoid Company, ON, Canada) supplemented with 5% sheep blood. The blood agar (BA) plates were incubated at 22°C for 24–48 h. Morphologically, the colonies separated well and appeared off-white with smooth edges on the culture plates after incubation. Non-distinct colonies with a mixture of bacterial growth were sub-cultured to BA plates to obtain pure colonies.

### Strain selection and identification for main spectral profile creation

One isolate of *Y. ruckeri* was randomly selected from the culture collection of the Atlantic salmon group (*n* = 27) for the creation of the MSP. The DNA of the selected isolate was extracted using the InstaGene™ Matrix (BioRad), following the manufacturer's guidelines with the following modifications: a loopful of bacteria instead of one colony was collected for the starting material, and the incubation time at 56°C was increased from 30 to 45 min. The 16S rRNA gene was amplified by PCR using primers 27F and 1492R (35), amplicons visualized on agarose gel, and sent to a commercial laboratory for purification and Sanger sequencing. Sequences were trimmed and aligned using CLC Sequence Viewer 7, and sequence identity was determined using the National Center Biotechnology Information nucleotide collection (BLASTn).

### Creation of main spectral profile for *Yersinia ruckeri*

The creation of MSP for *Y. ruckeri* was done following Bruker's recommended procedure for custom MALDI Biotyper^®^ (MBT) MSP and library creation.

#### Protein extraction procedure

Three hundred microliters of deionized water were measured into a 1.5 ml Eppendorf microcentrifuge tube using a pipette, followed by placing a single, fresh (24–48 h old), and pure colony of *Y. ruckeri* into the same tube. This was vortexed thoroughly and added a total of 900 μL of absolute ethanol to the mixture in the microcentrifuge tube. The mixture was centrifuged at a maximum speed of 13,000 revolutions per minute (rpm) for 2 min. The supernatant was decanted, while the residue in the tube was centrifuged again to separate any trace of absolute ethanol. The remaining ethanol was carefully decanted by using a pipette not to disturb the pellet formed at the bottom of the tube. The pellet was left to air dry at room temperature for at least 5 min. Once dried, 20 μL of freshly prepared 70% formic acid (Honeywell Fluka, NC, USA) was added to the pellet and vortexed. A volume of 20 μL of acetonitrile (ACN) (Sigma-Aldrich Canada Co., ON, Canada) was added to the mixture and placed in a centrifuge for 2 minutes at a maximum speed of 13,000 rpm. One microliter of supernatant was pipetted onto a clean MALDI stainless target plate and was repeated for ten replicates. The spots were allowed to air dry at room temperature. Within 30 min after drying, spots were overlaid with 1 μL of α-cyano-4-hydroxycinnamic acid (HCCA) matrix solubilized in 50% acetonitrile (Sigma-Aldrich Canada Co., ON, Canada), 47.5% water, and 2.5% trifluoroacetic acid solution. The matrix was left to air dry at room temperature.

#### Instrument calibration

One microliter of Bacterial Test Standard (BTS; Bruker) was applied to a clean spot on the same MALDI stainless steel target and allowed to air dry. After drying, the BTS spot was overlaid with 1 μL of HCCA matrix solution within 30 min and allowed to air dry at room temperature.

The target was loaded into the microflex^®^ LT mass spectrometer (Bruker) for spectral acquisition. A cumulative spectrum is generated using six sets of 40 laser shots for each spot (for a cumulative total of 240 shots per spot) with a nitrogen laser frequency of 60-Hz, a voltage of 20 kv, and mass to charge ratio of ions range of 2–20 k Dalton. The instrument was calibrated with the BTS using the AutoXecute algorithm in flexControl^®^ software version 3.4 (Bruker) to manufacturer specifications. For calibration, the automatic laser position was manually overridden by moving the laser to the left side of the spot. The instrument was considered to pass calibration with eight reference mass peaks successfully assigned within ± 300 parts per million.

#### Main spectral profile creation and quality control

Spectra were acquired using the same laser conditions as the BTS from each of 10 supernatant spots and repeated three times, resulting in a collection of 30 cumulative spectra. The acquired spectra were saved and analyzed using the flexAnalysis^®^ software version 3.4 (Bruker). Spectra underwent automatic smoothing and baseline subtraction. Visual assessments of spectra were performed to identify flatline spectra, outlier mass peaks, dramatic mass shifts, or anomalies. Flatline spectra and outliers were removed prior to MSP creation. Five of the 30 spectra were excluded as outliers (having non-uniform peaks). In comparison, 25 spectra were saved as the MSP to the reference library as “*Yersinia ruckeri* 15945-2008 AVC 2021”. Hereafter, the new library will be referred to as the “novel MSP.”

### Validation of novel main spectral profile

The remaining 29 isolates of *Y. ruckeri* are referred to as “validation set” hereafter, for ease of description, in this study. The direct smear method, in which a colony of *Y. ruckeri* is smeared onto the MALDI plate using a toothpick, then overlaid with 1 μL of HCCA matrix within 30 min, followed by instrument calibration, spectra collection, and analysis were carried out for the validation set. The acquired spectra from the validation set were carefully analyzed. The analyzed spectra were compared against the novel MSP and the MBT reference library (which contains Bruker MSPs) in AVC ADSBL. The reliability of using the novel MSP and Bruker MSPs was based on the log score values recommended by the manufacturer for the MBT Compass^®^ software: a log score of 2.0 to 3.0 indicates high confidence identification, 1.7–1.9 indicates low confidence identification, and log score value < 1.7 indicates no reliable identification.

Analytical sensitivities of MALDI-TOF MS using the novel MSP and the Bruker MSPs were estimated based on high confidence identification of *Y. ruckeri* (log score value ≥ 2.0) with 95% confidence intervals (CI) calculated using the exact method in Stata version 15.

One isolate from the validation set was randomly selected for repeatability testing. Thirty spots on the selected isolate were directly smeared on a MALDI target, followed by instrument calibration, spectral acquisition, and analysis. These procedures were performed by a single technician on the same day. The acquired spectra from each of the 30 spots were compared against the novel MSP to identify the isolate and evaluate the degree of variability in their log scores. The organism best matched and the log score of identification were recorded, and the mean, standard deviation, range, and coefficient of variation in the log score of the isolates obtained from 30 different spots on the plate were calculated using Stata version 15.

MSP dendrogram analysis was performed to determine the relatedness in the mass peptide fingerprint of the MBT reference library and the novel MSP using distance correlation and single linkage function in the MBT Compass Explorer. The analyzed spectra of the validation set were cross-validated with the MSPs of other *Yersinia* species (including *Y. intermedia, Y. massilensis, Y. bercovieri, Y. mollareti, Y. aleksiciae, Y. aldovae, Y. frederiksenii, Y. rohdei, Y. kristensenii, Y. enterocolitica* subsp. *palearctica, Y. enterocolitica* subsp. *enterocolitica*, and *Y. pseudotuberculosis*) in the MBT reference library. The analyzed spectra of the validation set were further cross-validated with the novel MSP and the MSPs of bacterium like *Aeromonas* spp. (common gram-negative bacteria of salmonids) and other closely related bacteria which belong to the order Enterobacterales, such as *Serratia* spp., *Hafnia* spp., and *Edwardsiella* spp. (all have cultural and morphological similarity to *Y. ruckeri*).

## Results

The identity of the isolate selected for creating the main spectral profile was verified using 16S rRNA gene sequencing. The isolate selected for MSP creation had a 99.13% identity to type strain *Yersinia ruckeri* DSM^®^ 18506™ (=ATCC^®^ 29473™) using a 1380 base-pair sequence and the GenBank accession number was KJ606914.1.

**Figure 1:**
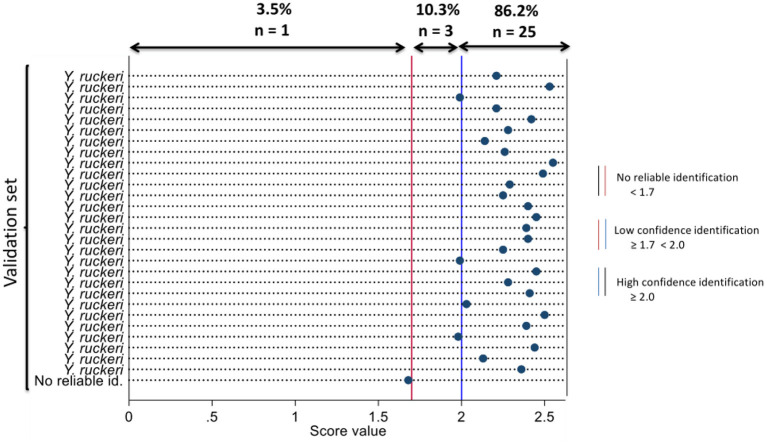
Dot diagram showing the organism that best matches the isolates in the validation set using the novel main spectral profile and their respective log scores at MALDI-TOF MS identification.

The novel main spectral profile was validated with acquired spectra from the validation set. The organisms that best match the validation set and log score values are represented using a dot map in Figure 1. Of the validation set compared against the novel MSP, 86.2% (*n* = 25) were identified as *Y. ruckeri* with high confidence (having log score values ≥ 2.0), 10.3% (*n* = 3) were identified as *Y. ruckeri* with low confidence (having log score values ≥ 1.7 < 2.0), and 3.5% (*n* = 1) was no reliable identification (having log score value < 1.7). Analytical sensitivity of MALDI-TOF MS using the novel MSP to identify the validation set was 86.2% (95% CI = 68.3–96.1) based on high confidence identification.

**Figure 2:**
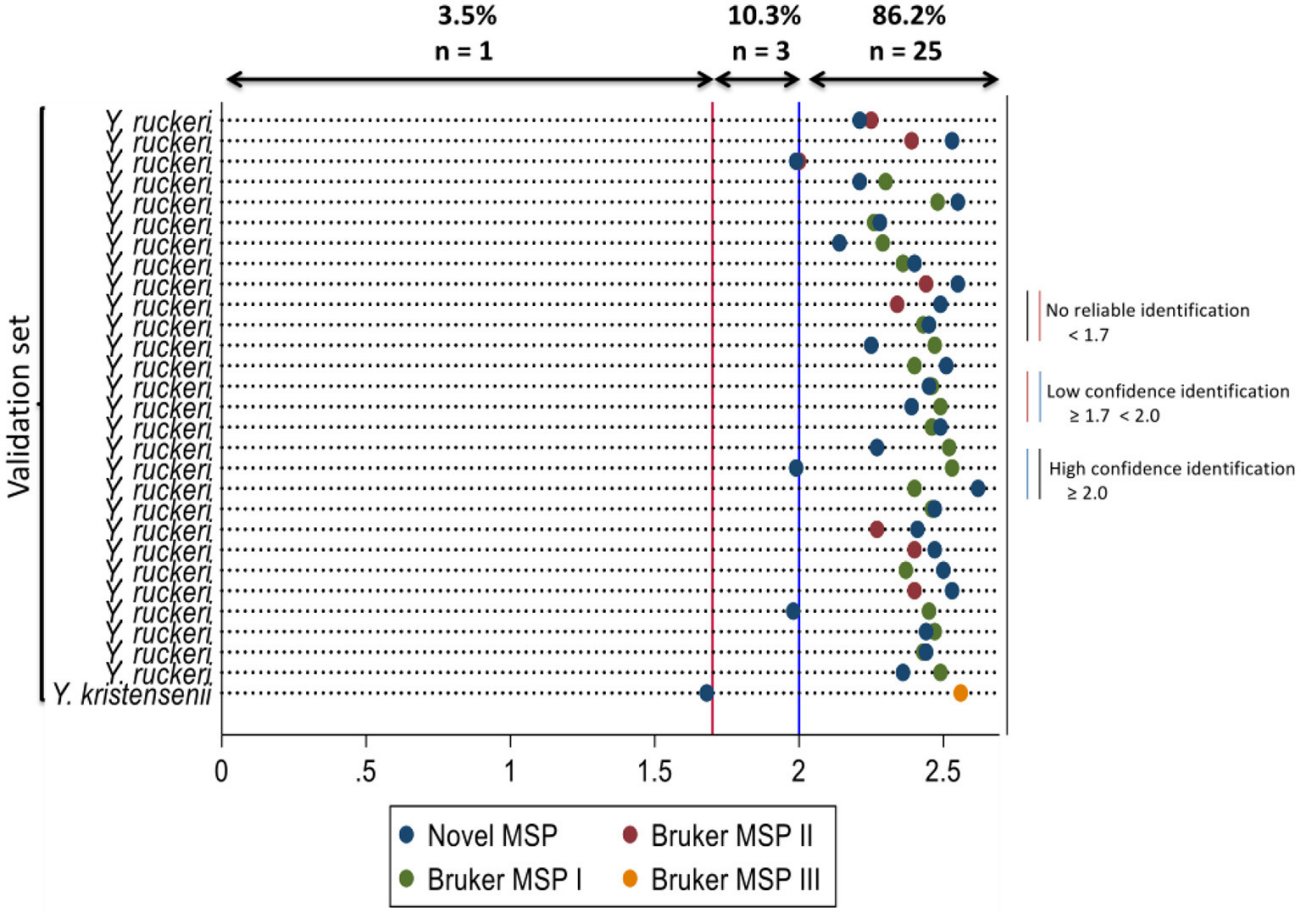
Dot diagram showing the organism that best matches the isolates in the validation set using the novel main spectral profile and the Bruker main spectral profiles with their respective log scores for MALDI-TOF MS identification. Novel MSP, *Yersinia ruckeri* 15945-2008 AVC 2021; Bruker MSP I, *Yersinia ruckeri* CCUG 21537 CCUG; Bruker MSP II, *Yersinia ruckeri* 3000 CVUA; Bruker MSP III, *Yersinia kristensenii* CCM 3562 CCM.

When the spectra of the validation set were compared against the MBT reference library and novel MSP, simultaneously; the novel MSP was the highest-ranked MSP which best identified *Y. ruckeri* isolates with high confidence for 16 individual isolates. The Bruker MSPs I and II were the highest-ranked MSPs that best identified *Y. ruckeri* isolates with high confidence for 10 and 2 individual isolates, respectively. The isolate classified as unreliable identification by the novel MSP was classified as *Y. kristensenii* with high confidence identification by the Bruker MSP III (Supplementary Figure 1). The log score values and organisms that best match the validation set using the Bruker MSPs and novel MSP at the MALDI-TOF MS identification are presented in Figure 2.

The species of fish from which the validation set was recovered, and the log score values obtained when the validation set were compared against the novel MSP are presented in (Supplementary Figure 2).

Repeatability testing of an isolate compared against the novel MSP yielded high confidence identification as *Y. ruckeri* for all the repeated runs (*n* = 30) with an average log score value of 2.38 (range, 2.2 to 2.6) from the 30 different spots of the isolate and has a low coefficient of variation (3.3%).

The novel MSP was clustered with the Bruker MSPs (*n* = 3) of *Y. ruckeri* and is clearly distinguished from other Bruker MSPs of other species of *Yersinia* (Supplementary Figure 3). During cross-validation of the validation set against MSPs of other *Yersinia* species (except *Y. ruckeri*) present in the MBT reference library, 96.6% (*n* = 28) were matched with other *Yersinia* species with low confidence identification. In contrast, an isolate from the validation set was classified as *Y. kristensenii* with high confidence identification (Supplementary Table 1). Cross-validation of the novel MSP, MSP of *Aeromonas* spp., MSPs of other close-related bacterial species such as *Serratia* spp., *Hafnia* spp., *Edwardsiella tarda*, and the validation set, yielded 89.7% (*n* = 26) high confidence identification of *Y. ruckeri*, 6.9% (*n* = 2) low confidence identification of *Y. ruckeri*, and 3.4% (*n* = 1) unreliable identification with the novel MSP only. In comparison, the validation set resulted in no reliable identification (100%, *n* = 29) when matched to MSPs of *Aeromonas spp., Serratia spp., Hafnia spp., and Edwardsiella tarda* (Supplementary Table 2).

## Discussion

This study describes the development of a main spectral profile (MSP) to rapidly identify *Yersinia ruckeri* isolated from salmonid samples using matrix-assisted laser desorption/ionization time-of-flight mass spectrometry (MALDI-TOF MS). This study has also provided the framework for MSP validation for other clinically important bacterial pathogens that affect salmonids aquaculture, especially in Canada, such as *Renibacterium salmoninarum, Aeromonas salmonicida*, and *Streptococcus iniae* (36).

The validation set used in this study was highly represented by *Y. ruckeri* isolates from Atlantic salmon than Atlantic cod and American eel. Furthermore, the novel MSP was developed using an isolate from the Atlantic salmon group because the novel MSP was primarily developed to identify *Y. ruckeri* in Atlantic salmon accurately. *Y. ruckeri* has been mostly detected from Atlantic salmon samples submitted for bacterial culture and isolation in the Atlantic Veterinary College, Aquatic Diagnostic Services Bacteriology Laboratory (AVC ADSBL) (7). However, the novel MSP successfully identified *Y. ruckeri* isolates from Atlantic salmon, Atlantic cod, and American eel in this study.

The direct smear method was used to validate the novel MSP as opposed to the protein extraction method because it is the same method used routinely by AVC ADSBL and most diagnostic labs for bacterial identification. The direct smear method is simpler to perform, faster, and it is not inferior to the protein extraction method for MALDI-TOF MS identification (37).

There are multiple MSPs available for identifying different bacterial species in the commercial reference library developed by Bruker to increase coverage of MALDI-TOF MS for identifying bacteria of various strains or serotypes (38, 39). In the present study, the analytical sensitivity of MALDI-TOF MS using the novel MSP provided high confidence identification for most of the *Y. ruckeri* isolates evaluated from Atlantic salmon. The novel MSP was the best to secure high confidence identification for 16 *Y. ruckeri* isolates evaluated compared to other individual MSPs developed by Bruker, which is in agreement with the report of Piamsomboon et al. (30). This finding gives credence to the extension or supplementation of the Bruker reference library with MSPs of aquatic origin to improve the identification of aquatic pathogens.

The *Y. ruckeri* isolate that could not be identified reliably with the novel MSP was identified as *Y. kristensenii* by Bruker MSP using the MALDI Biotyper^®^ (MBT) reference library and during cross-validation with other species of *Yersinia* (except *Y. ruckeri*). This suggests that the traditional identification method (including Analytical Profile Index^®^ 20E and or antisera agglutination method) used to confirm the validation set misclassified the isolate as *Y. ruckeri* and the Bruker MSP provided an accurate identification. Moreso, the isolate was detected and stored in 1990 in the AVC ADSL, and there is a possibility of misclassification, especially with the traditional identification methods. Gene sequencing of this isolate would have been recommended to provide a confirmatory identification; however, considering that the novel MSP classified the same isolate as no reliable identification twice, this was not pursued. Laboratories using traditional techniques to confirm bacteria not only stand the risk of bacterial misclassification at the species-level but also could overestimate or underestimate clinically relevant pathogens (40). On the other hand, misidentification of fish bacteria can have a severe economic impact. For instance, false-positive results may halt fish transfer from hatchery to production site and promote inappropriate use of antimicrobials for treatments which may lead to antimicrobial resistance (2). Misclassification error using the API 20E system for phenotypic identification of fish bacteria has been reported by several studies (41, 42). In addition, cross-reaction of bacterial isolates with antisera could potentially lead to bacterial misidentification (43). However, MALDI-TOF MS matches bacterial peptides from culture plates against spectra in the reference library, and the chance of misidentification is lower than the traditional method of bacterial identification (44).

Repeatability testing of a randomly-selected isolate against the novel MSP evaluated the variability in bacterial identification and the log score values. Each analyzed spectrum was correctly identified with high confidence as *Y. ruckeri*. The small variation in log scores from this analysis demonstrates a high confidence level in the result provided by the novel MSP. It should also be noted that sources of variation in log score could be from smear preparation and laser positioning, which might also explain why three of the validation set (*Y. ruckeri* from Atlantic salmon and American eel) evaluated were only identified with low confidence by the novel MSP.

During cross-validation, the novel MSP did not falsely identify any closely related bacterial species in the order Enterobacterales despite the relatedness, suggesting that the novel MSP was highly specific to identifying *Y. ruckeri* (45, 46). Furthermore, the MSP dendrogram clustered the novel MSP with Bruker MSPs of *Y. ruckeri*, and it clearly discriminates the collective MSPs of *Y. ruckeri* from others.

Individually, none of the Bruker MSPs were perfect to best identify all the isolates in the validation set evaluated in this study. It required three Bruker MSPs to identify the validation set with high confidence collectively. While it is prudent to supplement the Bruker reference library with new MSPs of aquatic pathogen, the novel MSP in this study has been uploaded and updated to the MBT reference library for rapid identification of *Y. ruckeri* from samples submitted for bacterial culture and isolation in AVC ADSBL and the novel MSP can be shared and used by other aquatic animal laboratories around the world.

## Conclusion

This study has developed and validated a protein spectrum for the rapid detection of *Yersinia ruckeri* in Atlantic salmon using the matrix-assisted laser desorption/ionization time-of-flight mass spectrometry.

## Study limitations

All the isolates in the validation set utilized for this study were not gene-sequenced because of cost implications. Molecular sequencing could have been used as a gold standard, and this would have identified the isolate in the validation set that was classified as *Y. kristensenii* by the Bruker MSP. In addition, strain diversity of the isolates used for creating the novel MSP and the validation set were unknown. This could have provided clearer insight into the serotype of *Y. ruckeri* identified by the novel MSP. Lastly, a single highly trained technician was used to conduct all experiments, and a repeatability assessment using two or more technicians in the same laboratory might have identified additional variability in results.

## Data availability statement

The original contributions presented in the study are included in the article/Supplementary material, further inquiries can be directed to the corresponding author/s.

## Author contributions

RO, IG, DG, MS, SS, and KT conceived the study idea and designed the experiment. RO and MS conducted the experiment. RO wrote the first draft of the manuscript. IG and KT supervised the work. All authors have read, edited, and approved the final manuscript.

## Conflict of interest

The authors declare that the research was conducted in the absence of any commercial or financial relationships that could be construed as a potential conflict of interest.

## Publisher's note

All claims expressed in this article are solely those of the authors and do not necessarily represent those of their affiliated organizations, or those of the publisher, the editors and the reviewers. Any product that may be evaluated in this article, or claim that may be made by its manufacturer, is not guaranteed or endorsed by the publisher.
