# Supplementary figures and images for: Development and validation of main spectral profile for rapid identification of Yersinia ruckeri isolated from Atlantic salmon using matrix-assisted laser desorption/ionization time-of-flight mass spectrometry
Source: Front Vet Sci. 2022 Oct 20;9:1031373. doi: 10.3389/fvets.2022.1031373 (PMC9630595; doi:10.3389/fvets.2022.1031373)

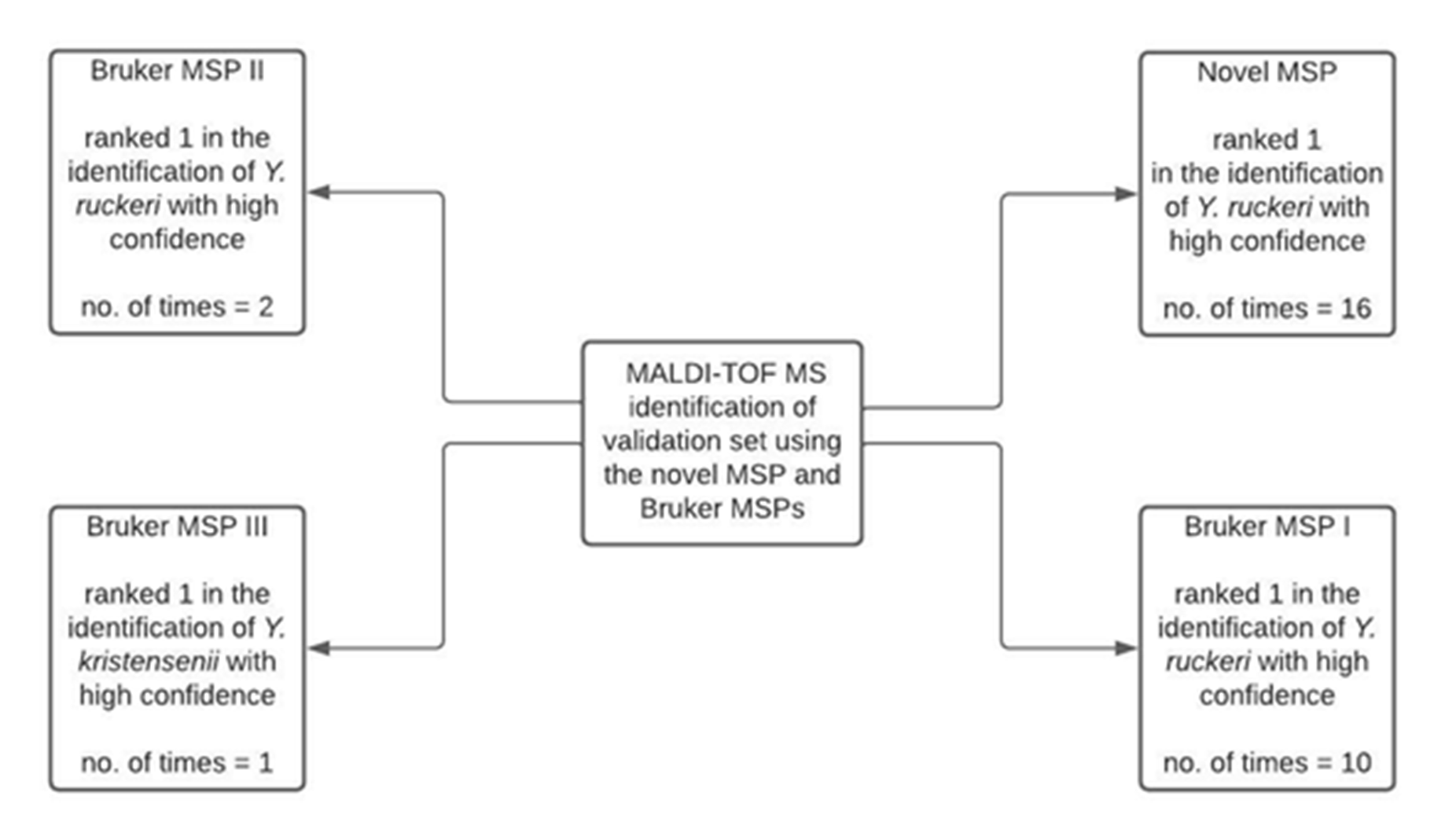

Supplement: Supplementary file 1 [file Image_1.tif]

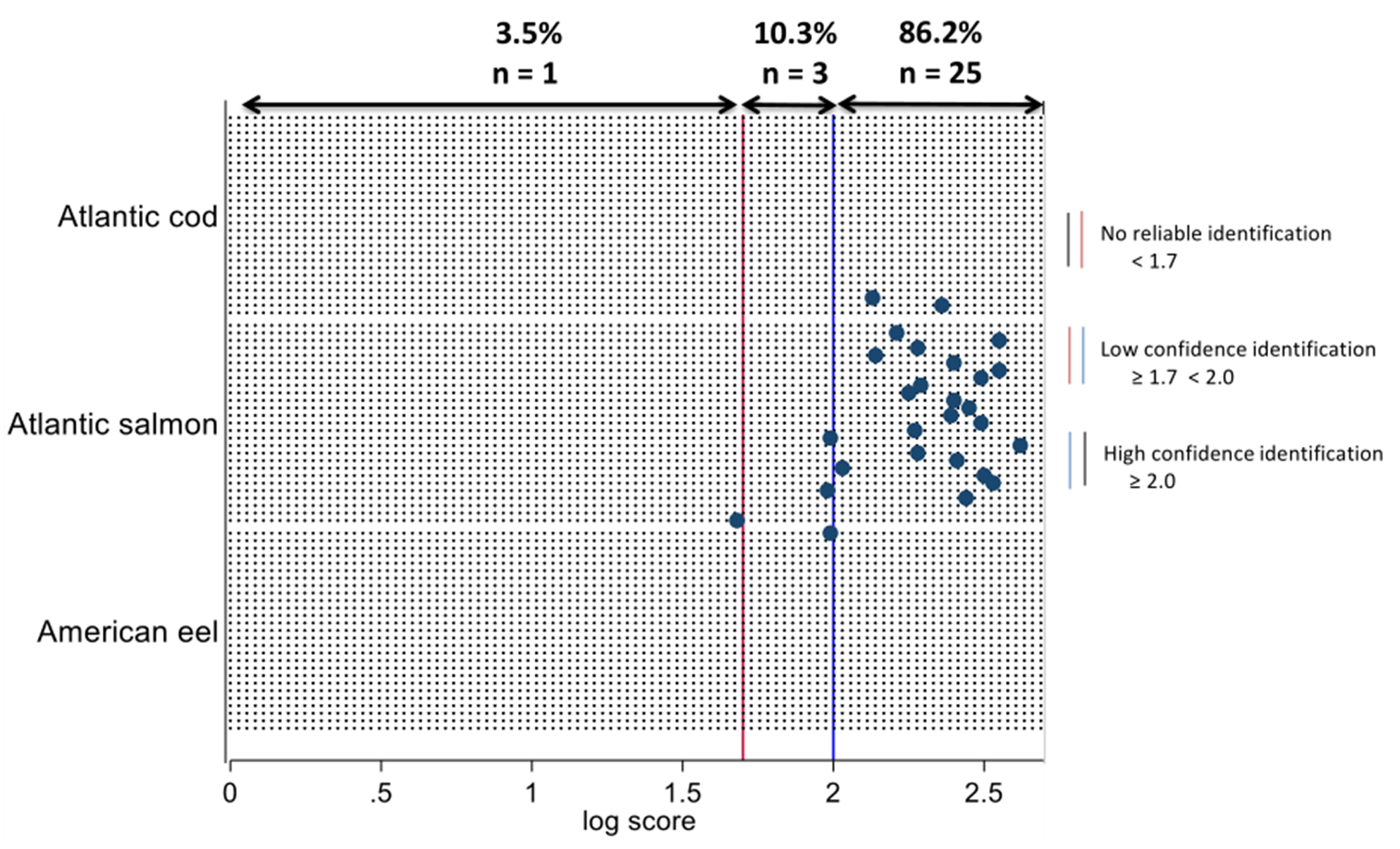

Supplement: Supplementary file 2 [file Image_2.tif]

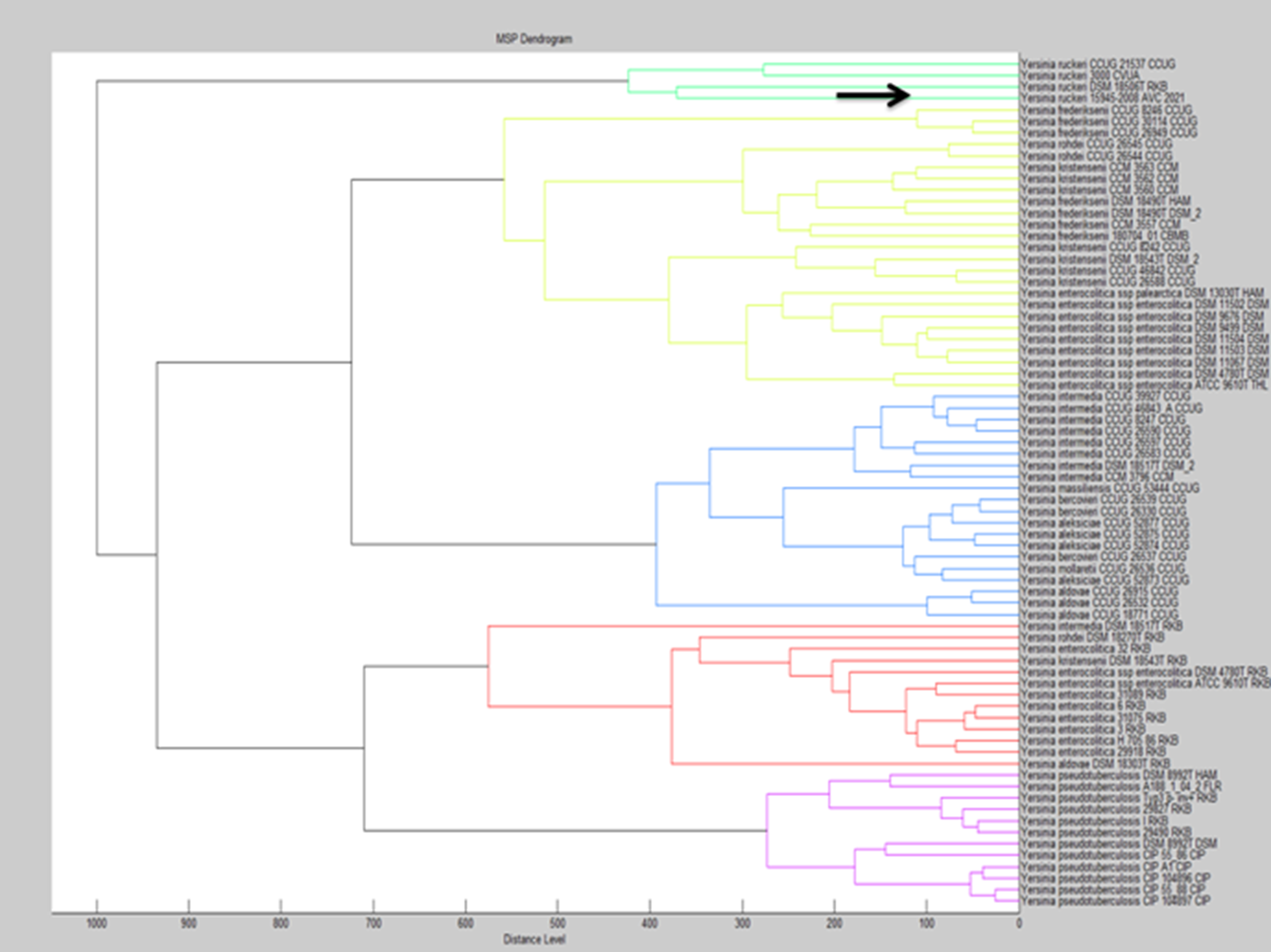

Supplement: Supplementary file 3 [file Image_3.tif]
