# Supplementary material for: Development and validation of main spectral profile for rapid identification of Yersinia ruckeri isolated from Atlantic salmon using matrix-assisted laser desorption/ionization time-of-flight mass spectrometry
Source: Front Vet Sci. 2022 Oct 20;9:1031373. doi: 10.3389/fvets.2022.1031373 (PMC9630595; doi:10.3389/fvets.2022.1031373)
Supplement: Supplementary file 4 [file Table_1.docx]

**Supplementary Table 1 |** Cross-validation of the validation set with main spectral profiles of other species of *Yersinia* excluding *Yersinia ruckeri* in the MALDI Biotyper® reference library

|  | **Bacterial isolates lab no.** | **Species** | **Organism best matched the MSPs of other species of *Yersinia*** | **Log score value / range of log score value** |
| --- | --- | --- | --- | --- |
| 1 | *Yersinia ruckeri* 25907-2008 | Atlantic salmon | *Yersinia intermedia*  *Yersinia frederiksenii* | 1.95  1.95 |
| 2. | *Yersinia ruckeri* | Atlantic salmon | *Yersinia pseudotuberculosis*  *Yersinia frederiksenii* | 1.75 – 1.77  1.94 |
|  | 14969-2010 |  | *Yersinia intermedia* | 1.89 |
|  |  |  | *Yersinia enterocolitica ssp enterocolitica* | 1.84 |
| 3. | *Yersinia ruckeri* | Atlantic salmon | *Yersinia pseudotuberculosis*  *Yersinia frederiksenii* | 1.88  1.73 |
|  | 16319-2009 |  | *Yersinia aleksiciae* | 1.83 |
|  |  |  | *Yersinia intermedia* | 1.80 |
| 4. | *Yersinia ruckeri* | Atlantic salmon | *Yersinia kristensenii*  *Yersinia frederiksenii* | 1.77  1.74 |
|  | 18169-2007 |  | *Yersinia intermedia* | 1.81 |
|  |  |  | *Yersinia pseudotuberculosis* | 1.86 – 1.95 |
|  |  |  | *Yersinia aleksiciae* | 1.79 – 1.89 |
| 5. | *Yersinia ruckeri* | Atlantic salmon | *Yersinia pseudotuberculosis*  *Yersinia frederiksenii* | 1.82 – 1.95  1.61 - 1.95 |
|  | 18170-2007 |  | *Yersinia intermedia* | 1.90 |
|  |  |  | *Yersinia aleksiciae* | 1.79 – 1.89 |
| 6. | *Yersinia ruckeri* | Atlantic salmon | *Yersinia pseudotuberculosis*  *Yersinia intermedia* | 1.86 - 1.96  1.90 |
|  | 19077-2011 |  | *Yersinia frederiksenii* | 1.95 |
|  |  |  | *Yersinia intermedia* | 1.85 – 1.91 |
|  |  |  | *Yersinia frederiksenii* | 1.87 |
| 8. | *Yersinia ruckeri* 21383-2008 | Atlantic salmon | *Yersinia intermedia*  *Yersinia kristensenii* | 1.81 – 1.88  1.58 – 1.59 |
| 9. | *Yersinia ruckeri* 23973-2013 | Atlantic salmon | *Yersinia aleksiciae*  *Yersinia pseudotuberculosis* | 1.86 – 1.93  1.83 – 1.90 |
| 10. | *Yersinia ruckeri* 24979-2011 | Atlantic salmon | *Yersinia kristensenii*  *Yersinia pseudotuberculosis*  *Yersinia enterocolitica ssp enterocolitica*  *Yersinia intermedia* | 1.79 – 1.90  1.76  1.91  1.88 – 1.92 |
| 11. | *Yersinia ruckeri* 27447-2011 | Atlantic salmon | *Yersinia intermedia*  *Yersinia enterocolitica ssp enterocolitica*  *Yersinia frederiksenii* | 1.85  1.79  1.79 – 1.87 |
| 12. | *Yersinia ruckeri* 28714-2006 | Atlantic salmon | *Yersinia kristensenii*  *Yersinia pseudotuberculosis*  *Yersinia frederiksenii* | 1.84 – 1.92  1.78 – 1.88  1.81 – 1.88 |
| 13. | *Yersinia ruckeri* 34698-2009 | Atlantic cod | *Yersinia pseudotuberculosis* | 1.88 – 1.98 |
| 14. | *Yersinia ruckeri* 35090-2009 | Atlantic cod | *Yersinia pseudotuberculosis*  *Yersinia intermedia*  *Yersinia frederiksenii*  *Yersinia enterocolitica ssp enterocolitica* | 1.84 – 1.88  1.84  1.87  1.75 – 1.83 |
| 15. | *Yersinia ruckeri* 11395-2010 | Atlantic salmon | *Yersinia kristensenii*  *Yersinia pseudotuberculosis* | 1.75  1.71 |
| 16. | *Yersinia ruckeri* 11714-2008 | Atlantic salmon | *Yersinia kristensenii* | 1.82 – 1.85 |
| 17. | *Yersinia ruckeri* 13631-2009 | American eel | *Yersinia enterocolitica ssp enterocolitica*  *Yersinia kristensenii* | 1.75  1.75 |
| 18. | *Yersinia ruckeri*  1463-2013 | Atlantic salmon | *Yersinia pseudotuberculosis*  *Yersinia aleksiciae* | 1.76  1.75 |
| 19. | *Yersinia ruckeri*  15945-2008 | Atlantic salmon | *Yersinia intermedia*  *Yersinia frederiksenii* | 1.93  1.92 |
| 20. | *Yersinia ruckeri*  16155-2003 | Atlantic salmon | *Yersinia frederiksenii*  *Yersinia aleksiciae* | 1.97  1.80 |
| 21. | *Yersinia ruckeri*  16743-2009 | Atlantic salmon | *Yersinia kristensenii*  *Yersinia pseudotuberculosis* | 1.82  1.82 |
| 22. | *Yersinia ruckeri*  18047-2008 | Atlantic salmon | *Yersinia enterocolitica ssp enterocolitica* | 1.77 – 1.81 |
| 23. | *Yersinia ruckeri*  19179-2012 | Atlantic salmon | *Yersinia enterocolitica ssp enterocolitica*  *Yersinia aleksiciae* | 1.93  1.88 |
| 24. | *Yersinia ruckeri*  19630-2004 | Atlantic salmon | *Yersinia intermedia* | 1.89 |
| 25. | *Yersinia ruckeri*  25932-2006 | Atlantic salmon | *Yersinia frederiksenii* | 1.77 – 1.79 |
| 26. | *Yersinia ruckeri*  27451-2011 | Atlantic salmon | *Yersinia intermedia*  *Yersinia frederiksenii* | 1.91  1.86 |
| 27. | *Yersinia ruckeri*  29112-2021 | Atlantic salmon | *Yersinia frederiksenii*  *Yersinia pseudotuberculosis* | 1.91  1.91 |
| 28. | *Yersinia ruckeri*  31718-2009 | Atlantic salmon | *Yersinia mollaretii*  *Yersinia frederiksenii* | 1.86  1.83 |
| 29. | ***Yersinia ruckeri***  **9275-1990** | Atlantic salmon | *Yersinia kristensenii*  *Yersinia enterocolitica ssp enterocolitica*  *Yersinia bercovieri* | 2.30 – 2.56  2.18 – 2.19  2.18 |

**NB:** Cell in bold represent bacterial isolate that was identified as no reliable identification by the novel main spectral profile in the validation set

**NB:** When the same bacteria is detected multiple times, the name of the bacterium and range of the log scores are presented
