# Supplementary material for: Development and validation of main spectral profile for rapid identification of Yersinia ruckeri isolated from Atlantic salmon using matrix-assisted laser desorption/ionization time-of-flight mass spectrometry
Source: Front Vet Sci. 2022 Oct 20;9:1031373. doi: 10.3389/fvets.2022.1031373 (PMC9630595; doi:10.3389/fvets.2022.1031373)
Supplement: Supplementary file 5 [file Table_2.docx]

**Supplementary Table 2 |** Cross-validation of the validation set, novel MSP, MSP of *Aeromonas* spp., and MSPs of closely related bacterial species to *Yersinia ruckeri*

|  | **Bacterial isolates lab no.** | **Species** | **Organism**  **(best matched the novel MSP)** | **Log score value** | **Organism**  **(best matched the MSPs**  **of *Aeromonas* spp.,**  ***Serratia* spp., *Hafnia* spp.,**  ***Edwardsiella* spp.)** | **Log score value** |
| --- | --- | --- | --- | --- | --- | --- |
| 1. | *Yersinia ruckeri*  21383-2008 | Atlantic salmon | *Yersinia ruckeri* | 2.04 | No reliable identification | 1.34 |
| 2. | *Yersinia ruckeri*  23973-2013 | Atlantic salmon | *Yersinia ruckeri* | 2.22 | No reliable identification | 1.59 |
| 3. | *Yersinia ruckeri*  35090-2009 | Atlantic cod | *Yersinia ruckeri* | 2.26 | No reliable identification | 1.34 |
| 4. | *Yersinia ruckeri*  13631-2009 | American eel | *Yersinia ruckeri* | 1.99 | No reliable identification | 1.12 |
| 5. | *Yersinia ruckeri*  14969-2010 | Atlantic salmon | *Yersinia ruckeri* | 2.55 | No reliable identification | 1.64 |
| 6. | *Yersinia ruckeri*  16319-2009 | Atlantic salmon | *Yersinia ruckeri* | 2.40 | No reliable identification | 1.41 |
| 7. | *Yersinia ruckeri*  18169-2007 | Atlantic salmon | *Yersinia ruckeri* | 2.41 | No reliable identification | 1.47 |
| 8. | *Yersinia ruckeri*  18170-2007 | Atlantic salmon | *Yersinia ruckeri* | 2.34 | No reliable identification | 1.47 |
| 9. | *Yersinia ruckeri*  19077-2011 | Atlantic salmon | *Yersinia ruckeri* | 2.51 | No reliable identification | 1.61 |
| 10. | *Yersinia ruckeri*  20231-2006 | Atlantic salmon | *Yersinia ruckeri* | 2.49 | No reliable identification | 1.49 |
| 11. | *Yersinia ruckeri*  9275-1990 | Atlantic salmon | No reliable identification | 1.68 | No reliable identification | 1.44 |
| 12. | *Yersinia ruckeri*  15945-2008 | Atlantic salmon | *Yersinia ruckeri* | 2.28 | No reliable identification | 1.42 |
| 13. | *Yersinia ruckeri*  24979-2011 | Atlantic salmon | *Yersinia ruckeri* | 2.62 | No reliable identification | 1.39 |
| 14. | *Yersinia ruckeri*  25907-2008 | Atlantic salmon | *Yersinia ruckeri* | 2.47 | No reliable identification | 1.57 |
| 15. | *Yersinia ruckeri*  27447-2011 | Atlantic salmon | *Yersinia ruckeri* | 2.44 | No reliable identification | 1.46 |
| 16. | *Yersinia ruckeri*  28714-2006 | Atlantic salmon | *Yersinia ruckeri* | 2.51 | No reliable identification | 1.51 |
| 17. | *Yersinia ruckeri*  34698-2009 | Atlantic Cod | *Yersinia ruckeri* | 2.44 | No reliable identification | 1.39 |
| 18. | *Yersinia ruckeri*  11395-2010 | Atlantic salmon | *Yersinia ruckeri* | 2.21 | No reliable identification | 1.41 |
| 19. | *Yersinia ruckeri*  11714-2008 | Atlantic salmon | *Yersinia ruckeri* | 2.53 | No reliable identification | 1.49 |
| 20. | *Yersinia ruckeri*  31718-2009 | Atlantic salmon | *Yersinia ruckeri* | 2.44 | No reliable identification | 1.66 |
| 21. | *Yersinia ruckeri*  1463-2013 | Atlantic salmon | *Yersinia ruckeri* | 2.21 | No reliable identification | 1.42 |
| 22. | *Yersinia ruckeri*  16155-2003 | Atlantic salmon | *Yersinia ruckeri* | 2.14 | No reliable identification | 1.46 |
| 23. | *Yersinia ruckeri*  16743-2009 | Atlantic salmon | *Yersinia ruckeri* | 2.55 | No reliable identification | 1.57 |
| 24. | *Yersinia ruckeri*  18047-2008 | Atlantic salmon | *Yersinia ruckeri* | 2.49 | No reliable identification | 1.45 |
| 25. | *Yersinia ruckeri*  19179-2012 | Atlantic salmon | *Yersinia ruckeri* | 2.45 | No reliable identification | 1.42 |
| 26. | *Yersinia ruckeri*  19630-2004 | Atlantic salmon | *Yersinia ruckeri* | 2.39 | No reliable identification | 1.51 |
| 27. | *Yersinia ruckeri*  25932-2006 | Atlantic salmon | *Yersinia ruckeri* | 2.41 | No reliable identification | 1.47 |
| 28. | *Yersinia ruckeri*  27451-2011 | Atlantic salmon | *Yersinia ruckeri* | 2.50 | No reliable identification | 1.52 |
| 29. | *Yersinia ruckeri*  29112-2021 | Atlantic salmon | *Yersinia ruckeri* | 1.98 | No reliable identification | 1.64 |
